# A Risk Assessment Tool for Resumption of Research Activities During the COVID-19 Pandemic

**DOI:** 10.21203/rs.3.rs-103997/v1

**Published:** 2020-11-12

**Authors:** Suzanne M Simkovich, Lisa M. Thompson, Maggie Clark, Kalpana Balakrishnan, Alejandra Bussalleu, William Checkley, Thomas Clasen, Victor Davila-Roman, Anaite Diaz-Artiga, Lisa de las Fuentes, Steven Harvey, Miles Kirby, Amy Lovvorn, Eric McCollum, Jennifer Peel, Ashlinn Quinn, Ghislaine Rosa, Lindsay Underhill, Kendra Williams, Bonnie Young, Joshua Rosenthal

**Affiliations:** MedStar Health Research Institute; Emory University Nell Hodgson Woodruff School of Nursing; Colorado State University; Sri Ramachandra Institute for Higher Education and Research; Universidad Peruana Cayetano Heredia; Johns Hopkins: Johns Hopkins University; Emory University School of Public Health; Washington University In St Louis: Washington University in Saint Louis; Universidad del Valle de Guatemala; Washington University In St Louis: Washington University in Saint Louis; Johns Hopkins University; Harvard University; Emory University; Johns Hopkins: Johns Hopkins University; Colorado State University; National Institutes of Health; London School of Hygiene & Tropical Medicine; Johns Hopkins: Johns Hopkins University; Johns Hopkins: Johns Hopkins University; Colorado State University; National Institutes of Health

**Keywords:** Risk Assessment, Biosafety, Research, COVID-19, SARS-CoV-2

## Abstract

**Rationale:**

The spread of severe acute respiratory syndrome coronavirus-2 has suspended many non-COVID-19 related research activities. Where restarting research activities is permitted, investigators need to evaluate the risks and benefits of resuming data collection and adapt procedures to minimize risk.

**Objectives:**

In the context of the multicountry Household Air Pollution Intervention (HAPIN) trial, we developed a framework to assess the risk of each trial activity and to guide protective measures. Our goal is to maximize integrity of reseach aims while minimizing infection risk based on the latest understanding of the virus.

**Methods:**

We drew on a combination of expert consultations, risk assessment frameworks, institutional guidance and literature to develop our framework. We then systematically graded clinical, behavioral, laboratory and field environmental health research activities in four countries for both adult and child subjects using this framework.

**Results:**

Our framework assesses risk based on staff proximity to the participant, exposure time between staff and participants, and potential aerosolization while performing the activity. One of of four risk levels, from minimal to unacceptable, is assigned and guidance on protective measures is provided. Those activities which can potentially aerosolize the virus are deemed the highest risk.

**Conclusions:**

By applying a systematic, procedure-specific approach to risk assessment for each trial activity, we can compare trial activities using the same criteria. This approach allows us to protect our participants and research team and to uphold our ability to deliver on the research commitments we have made to our participants, local communities, and funders.

## Background

The spread of severe acute respiratory syndrome coronavirus-2 (SARS-CoV-2) and resulting coronavirus disease 2019 (COVID-19) has led to the temporary suspension of many non-COVID-19 related research activities worldwide. Where feasible, studies are considering remote data collection by telephone or web-based conferencing.[1–3] However, this approach is often not possible when performing anthropomorphic measurements, specimen collection or when investigators need to make other direct observations. Even temporary suspension of research activities can potentially cause harm if investigators are evaluating an intervention that is hypothesized to be beneficial. Further, the suspension of data collection could result in loss of study power and potentially introduce bias. Every day, we are gaining a greater understanding of the transmissibility of SARS-CoV-2, and this knowledge increases our ability to safely resume a wide variety of non-COVID-19 related research activities.[3, 4] Where local law or institutional regulations allow activities to restart, investigators need to evaluate the risks and benefits to both research staff and participants of resuming data collection. To safely conduct study activities, researchers need to develop standardized procedures that are based on realistic assessment of these risks, provide guidance on where and when they are manageable as well as how to minimize the risk with physical distance measures and appropriate personal protective equipment (PPE).

Investigators in the Household Air Pollution Intervention Network (HAPIN) trial initially suspended data collection due to the pandemic and have since restarted collection of behavioural, environmental, biological and clinical measurements in the fifth year of a five-year, multi-country trial.[5–8] HAPIN is a randomized controlled trial in Guatemala, India, Peru, and Rwanda that is assessing the health benefits of providing liquefied petroleum gas (LPG) stoves and an 18-month supply of free LPG to 3,200 households that otherwise depend on solid biomass fuel (wood, animal dung, or crop residue) for cooking. Measurements of cooking behavior, personal and in-home exposure to air pollution, biological samples and clinical measurements are being collected longitudinally from pregnant women and their newborns in every household, along with an older adult woman, if present.[5–8] Our study involves home visits, as well as visits to health centers and hospitals during the woman’s pregnancy and the first year of the child’s life.

As SARS-CoV-2 spread globally, all four countries’ governments implemented public safety restrictions that limited activities to those designated as essential. Essential activities varied across settings and at that time, research activities were not considered essential. However, LPG delivery for cooking was considered essential in all four countries. In Guatemala and Rwanda, our research teams were permitted to continue delivering LPG to study households without disruption. In India, the gas companies continued to deliver refill tanks to study participants. In Peru, our team was limited in its ability to deliver gas during the initial weeks of the restrictions. We were later able to re-establish services with a local gas company for delivery.

With the allowance for continued delivery of the LPG intervention, we immediately implemented changes in our delivery protocols to minimize SARS-CoV-2 risk. Further, in anticipation of the additional easement of movement restrictions in countries around the world, we developed a risk assessment tool with the guiding principle of ethical research to minimize the potential risks to research staff, participants and the community. We wanted our tool to be able to assess the risk of each study activity for each group of participants utilizing the same criteria, which is important in a large trial where there are multiple competing activities. Although the tool, using standardized definitions and criteria, has been designed within the framework of specific activities of the HAPIN trial, we report here on our approach, which we believe will be useful for other research contexts and questions.

## Methods

In developing our risk assessment tool, we drew on a combination of expert consultations, national and local expertise, institutional guidance and review of emerging literature.[9–17] We convened a multi-national panel of scientists and field team leaders from across the trial with expertise in the disciplines of clinical medicine and imaging, nursing, environmental science, epidemiology, behavioral science, community engagement and statistics, along with the trial funders who provide scientific guidance to the HAPIN trial. We sought input from local community leaders, the Ministries of Health, universities and non-governmental organizations regarding appropriate operations and safety concerns. We consulted with the Institutional Review Boards (IRBs) and Data and Safety Monitoring Board (DSMB) presiding over the trial regarding resumption of activities. We drew on historical occupational health frameworks for infectious disease biosafety and risk assessment and the most recent peer reviewed and grey literature about infection dynamics, as well as staff experience, to build a framework to evaluate risk of exposure to SARS-CoV-2.[3, 14, 18, 19] The intent was to develop criteria that were clear, simple and actionable for field managers and staff to implement and recommend appropriate practices and materials, in accordance with the risk level of each procedure and perceived risk threshold.

While SARS-CoV2 research findings are still changing, our assessment is based on the emerging consensus that aerosolization and droplet carriage of virus, primarily from coughing, sneezing, singing, crying, talking, and procedures such as swabbing the mouth or nose, are the predominant mode of infection.[20] It is unclear how long the virus remains in the air.[21–23] Fomites from surface contact may also contribute to transmission, but are likely a smaller risk.[24, 25] Evidence of SARS-CoV-2 presence has been detected in urine, stool, breast milk, semen and blood, but we are not aware of documented transmission through such specimens at the time of this publication.[21,26–29] Furthermore, the risk of transmission is greatest in the two days preceding onset of symptoms and continues afterward for at least ten days, and up to twenty days in immunosuppressed patients.[12, 13] Because documented asymptomatic carriage has been widely reported, we assumed that any staff member, collaborator or community participant might be shedding the virus.[30] Small children (especially infants) appear to be infected at the same rate as adults, but have more mild disease and thus may be unknowingly spreading disease. [31] We agreed that because viral transmissibility and the true prevalence of COVID-19 are not clearly known in any of our study sites due to limited testing, we erred on the side of caution and assumed moderate to substantial incidence of disease in most settings. This risk is defined as large-scale, uncontrolled or controlled community transmission. [32]

We assessed each HAPIN data collection activity among each group of participants (pregnant woman, infant, non-pregnant adult woman) because the risks may vary with each age group. Data collection activities were graded and agreed upon by the team of scientists. Local site investigators were asked to report perceived concerns by staff and participants in their communities. Risk factors and definitions were presented to the HAPIN steering committee for feedback before adoption. Standard Operating Procedures were developed for the resumption of study activities and included guidance on screening staff and participants for Covid-19 symptoms, transporting staff in project vehicles, cleaning equipment and surfaces, conducting home visits and health facility survelliance, and quarantining for suspected exposures to the virus. These documents are modified monthly to reflect the most up to date knowledge.

## Results

Risk criteria for each procedure included the age of participant, location, physical proximity necessary, exposure time, aerosolization potential, and criteria for use of available PPE (Table 1). Using these criteria we established a four level schedule for minimal to unacceptably high risk (Table 2). [14] We then proceeded to assess each research activity in detail according to the criteria outlined in Table 1 and assigned a risk level and appropriate PPE to each of these. Research activities assessed include fuel delivery, surveys, and data downloads, exposure assessment, biological sample collection and processing, clinical measures and observations in pregnant women, children, and vascular procedures in adults (Supplement).

Protective measures available in our settings are: a) where feasible, data collection is to be completed by telephone; b) where face to face activities can be conducted outside, they should be; c) when inside homes, clinics or offices staff and participants must minimize number of people in the room; d) rigourous hygiene for staff, materials, equipment and surfaces must be employed at all times; e) appropriate PPE is to be used based on the context and activity; f) under very high risk conditions the visit or the procedure should be suspended.

Using this assessment and taking necessary measures for protection, almost all of our research activities are deemed to pose potentially manageable risks. Biological sample collection spanned a range of assigned risk due to differences in participant-staff interaction. The activities with the highest level of risk were those that potentially aerosolize the virus during the procedure. For example, urine collection requires minimal contact (i.e., field workers instruct the participant to collect and store the urine sample until it can be retreived) resulting in low risk to both the participant and the study staff. However, dried blood spot collection from infants (who are unable to wear a mask and often cry during the procedure) could feasibly put field workers at high risk. To illustrate, examples of two procedures are provided in Table 1 and in context of all procedures in Table 5a of the supplement. We also identified several activities that are not part of our protocol, but that we believe would pose unmanageable risks (e.g. bronchoscopy, sputum inducting procedures, cardiopulmonary resusitation) for routine research in the pandemic context (Table 2).

## Discussion

Our risk assessment framework and semi-quantitative risk schedule allows flexibility to adjust risks and definitions as new evidence emerges about virus transmission. We believe that the approach and tools we summarize here can be adapted and will be of value as investigators assess and manage the risk of conducting their own research during the coronavirus pandemic. However, prior to the deployment of tools such as ours, researchers, in association with community members, IRBs, DSMBs, and funders need to evaluate the importance of any activity related to the primary aims of a trial against the associated risk. Necessarily, local health regulations related to mobility, home or clinic visits by researchers may supersede any of these judgments.

While this tool focuses on risk, decisions on what research should be continued in the presence of risk also require a careful assessment of benefits. After some discussion, we chose to make the risk-benefit calculation and decisions regarding which activities to suspend a secondary process because it requires a more complex judgment with potentially more subjectivity across investigators and local staff. Among the criteria we used to examine potential benefits of risky activities are whether or not the aim of any given procedure is to support a primary vs secondary or tertiary (exploratory) outcome of the trial protocol. Similarly, ancillary studies can potentially present additional risks above and beyond the primary outcomes. Further, if a measure can be eliminated in favor of a slightly less risky measure toward the same outcome we chose the former. For example, the Steering Committee has identified certain activities that are high risk (level 3) and, under the strain of the current conditions, may compromise the integrity of the trial’s main goals and relevant data obtainable under these procedures can be obtained through lower risk methods (e.g. oral rinse and venous blood draw – level 2)(Table 5a of the online supplement). Therefore, these activities have been suspended indefinitely (e.g., nasal brush and buccal scrape) and will only be reassessed as conditions change.

At the beginning of the pandemic, we temporarily suspended all activities except for fuel delivery until risk of the measurements could be assessed and procedures put into place to ensure safety. We continued collecting data by telephone when possible. When in-person contact was permitted by local IRBs, we used our framework to guide the appropriate protocols. During home visits, we provide adult participants and family members with paper or cloth masks to wear. Study staff maintain at least a two-meter distance between themselves and participants when collecting survey data, and only approach participants for hands-on activities such as blood pressure measurement, certain biological samples, or placing personal air pollution monitoring equipment. We attempt to perform as many procedures as possible outdoors. Participants are screened prior to arrival to the home for symptoms of COVID-19 and staff are screened daily and asked to stay home if there is any concern for COVID-19. When this has occurred, we have followed local guidance on when the quarantine period is complete. Staff regularly wash their hands with soap and water, and bring containers of water to house visits if water is not available, antibacterial hand gel is also used before and after each collection and house visit. If designated PPE is not available or cannot be used properly at any time, we postpone the activity. Similarly, our rules require goggles or a face shield for certain procedures, but participants (especially children) may find these terrifying, especially when combined with masks and gloves. In these situations, it may not be possible to complete the work as planned, and local staff have the autonomy and responsibility to decide whether any activity should proceed. Our guidance is based on expert opinion and has not been empirically verified at this time.

All of the above are taking place in the dynamic context of this pandemic. We plan to reassess each activity using our tool at least monthly as more information about SARS-CoV-2 transmission and the local epidemics becomes available. While the pandemic has been disruptive to our research, we believe there may also be some benefits from the shift in some data collection methods. For example, collecting data via telephone instead of visiting in-person increases time use efficiency for staff and decreases the burden of household visits on participants. Costs are lower, with less fuel used to travel via truck or motorbike to distant participant homes. On the other hand, telephone surveys may introduce uncertainty, if questions are complex in nature and may lead to poor response rates or lower quality data. We acknowledge that we have been able to resume study activities in some of our research sites, and attribute this to building relationships with participants over the past several years and the commitment of our local teams and collaborating institutions.

## Conclusion

We are optimistic that by applying this systematic, procedure-specific approach to risk assessment for each research activity, we will minimize the disruption in our trial due to the pandemic. While no activity in the current context is completely without risk of infection, we believe that this will support the completion of our primary research outcomes, and most importantly, protect our research staff and participants. In doing so, we aspire to comply with our ethical obligations to participants who agreed to participate in this trial, and to the communities and funders that have supported our work.

## Supplementary Material

Supplement

Supplement

## Figures and Tables

**Table 1:** Risk Assessment Framework

Risks	Definitions		Example 1-Lung Ultrasound Obtainment	Example 2-Personal Exposure Assessment

Participant	Participant group (i.e. pregnant woman/new mother, child, older adult woman)		Child	Older Adult Woman, Pregnant Woman

Location	Place where sample is collected or procedure performed		Healthcare facility	Home (indoor or outdoor)

Proximity to the participant	*Close*: The procedure requires the field worker and participant to be closer than 2 meters (6 feet) of one another.[11] If a procedure produces aerosolization, then it is automatically considered close contact.		Close	Close
	*Socially distant*: The procedure to be performed allows the field worker and participant to maintain a distance of > 2 meters (6 feet) apart from one another.			

Exposure time	*Short* : The procedure can be performed without the staff and participant to be in close proximity for > 15 minutes.[11]		Prolonged	Setup: short to prolonged
	*Prolonged*: The procedure requies the field worker and participant to be in close proximity for > 15 minutes.			Take-down: short

Aerosolization Potential	*None*: The procedure is unlikely to produce any aerosolized particles.		Yes	None
	*Yes*: The procedure may produce aerosolized particles.[11]			

Personal Protective Equipment (PPE) Needs	Criteria to determine PPE	PPE Needs	N95 or equivalent respirator + eye protection + gown + gloves	Paper facemask + eye protection + gloves

	Participant and staff are not in close contact at anytime. No aerosolizing procedures. No processing of biologic samples.	Paper or cloth facemask [9]		

	Participant and staff may be in close contact but only for a short period of time. Biologic materials may be processed in the lab. No aerosolizing procedures performed.	Paper facemask (preferably procedural quality) + eye protection + gloves (if the procedure requires touching the participant and/or a clinical specimen is collected)[9]		

	Participant and staff may be in close contact for a prolonged period of time and/or an aerosolizing procedure is occuring	N95 or equivalent respirator + eye protection + gown + gloves [9]		

	Participant and staff may be in close contact for a prolonged period of time and/or an aerosolizing procedure is occuring in a manner that staff and participants can not be safely protected.	Procedure will not be performed		

**Table 2:** Semi-quantitative risk schedule

Scale	Descriptor	Definition	Example (see Online Supplemental information for full descriptions)
Level 1	Minimal Risk	Participant and staff are not in close contact at anytime. No aerosolizing procedures. No processing of biologic samples.	Data collection by phone, in-person survey administration, fuel delivery
Level 2	Moderate Risk	Participant and staff may be in close contact but only for a short period of time. Biologic materials may be processed in the lab. No aerosolizing procedures performed.	Brachial artery reactivity testing, carotid artery reactivity testing, blood pressure measurement, fetal ultrasound, personal exposure assessment in adults, blood collection in adults, urine collection in adults
Level 3	High Risk	Participant and staff may be in close contact for a prolonged period of time or an aerosolizing procedure is occuring	Anthropometry, collection of blood in children, screening children for pneumonia, lung ultrasound, buccal scrape, nasal brush
Level 4	Unnaceptable Risk for Research	Participant and staff may be in close contact with patient samples for a prolonged period of time and an aerosolizing procedure is occuring in a manner that staff and participants can not be safely protected.	Bronchoscopy, Induced sputum, Cardiopulmonary resuscitation (CPR)

## Abbreviations

SARS-CoV-2: Severe acute respiratory syndrome coronavirus-2
COVID-19: Coronavirus disease 2019
PPE: Personal protective equipment
HAPIN: Household Air Pollution Intervention Network
IRBs: Institutional Review Boards
DSMB: Data and Safety Monitoring Board
