# Supplementary material for: A Risk Assessment Tool for Resumption of Research Activities During the COVID-19 Pandemic
Source: Res Sq. 2020 Nov 12:rs.3.rs-103997. Preprint. [Version 1] doi: 10.21203/rs.3.rs-103997/v1 (PMC7668754; doi:10.21203/rs.3.rs-103997/v1)
Supplement: Supplement [file 1b47eb653356210d1fc01ac5.docx]

Online Data Supplement

| Table 3. Materials Delivery, Surveys, Data-downloads | | | | | |
| --- | --- | --- | --- | --- | --- |
|  | Fuel Delivery | Survey Administration | | Stove Use Monitoring | |
| Risks | LPG Tank swap | In-person | Over the phone | Indoor SUMS Download | Outdoor SUMS Download |
| Participant | *Pregnant woman, older woman* | *Pregnant woman, adult woman* | *Pregnant woman, Older adult woman* | NA | NA |
| Location of collection | Home | Home | Office | Home (indoor) | Home (outdoor) |
| Proximity to the participant | Socially distant | Socially distant | Socially distant | Socially distant | Socially distant |
| Exposure time | Short | Short | None | Setup: none  Take-down: none | Setup: none  Take-down: none |
| Aerosolization | None | No | No | None | None |
| PPE Needs | Paper or cloth facemask | Paper or cloth facemask | Paper or cloth facemask | Setup & take-down: Paper or cloth facemask | Setup & take-down: Paper or cloth facemask |
| Overall Risk Score | 1 | 1 | 1 | 1 | 1 |

| Table 4a. Exposure Assessment in homes | | | | | |
| --- | --- | --- | --- | --- | --- |
| Risks | Personal Exposure | Personal Exposure | Indoor Area Sampling | Outdoor Area Sampling | Ambient Sampling |
| Participant | Older Adult Woman, Pregnant Woman | Child | NA | NA | NA |
| Location of collection | Home (indoor or outdoor) | Home (indoor or outdoor) | Home (indoor) | Home (outdoor) | Health Facility (setup outdoors but requires entering indoor areas) |
| Proximity to the participant | Close | Close | Socially distant | Socially distant | Socially distant |
| Exposure time | Setup: short to prolonged  Take-down: short | Setup: short  Take-down: short | Setup: none  Take-down: none | Setup: none  Take-down: none | Setup: none  Take-down: none |
| Aerosolization | None | None | None | None | None |
| PPE Needs | Setup & take-down: Paper/ cloth facemask/ + eye protection + gloves | Setup & take-down:  Paper/ cloth facemask + eye protection | Setup & take-down: Paper or cloth facemask | Setup & take-down: Paper or cloth facemask | Setup & take-down: Paper or cloth facemask |
| Overall Risk Score | 2 | 2 | 1 | 1 | 1 |
| Table 4b. Exposure Assessment in Lab | | | | | |
| Risks | Data Download | PM Filter handling | PM Filter weighing | BC Optical Assesments |  |
| Aerosolization | None | None | None | None |  |
| PPE Needs | Paper facemask | Paper facemask | Paper facemask | Paper facemask |  |
| Overall Risk Score | 1 | 1 | 1 | 1 |  |
| *All lab procedures are performed by the staff member without physical contact with a participant or other staff member. | | | | | |

| Table 5a. Biomarkers - Sample Collection | | | | | | | | |
| --- | --- | --- | --- | --- | --- | --- | --- | --- |
| Risks | Urine – main study | Whole Blood (finger stick dried blood spots)- main study | Whole Blood (finger stick dried blood spots)- main study | Whole Blood (venous blood draw)- *NCI substudy* | Buccal cells (buccal scrape)- *NCI substudy* | Buccal cells (oral rinse)- *NCI substudy* | Nasal cells (nasal brush)- *NCI substudy* | Stool (diaper)– *ancillary study* |
| Participant | *Child* | *Adult* | *Child* | *Adult* | *Adult* | *Adult* | *Adult* | *Child* |
| Location of collection | Home | Home | Home | Home | Home | Home | Home | Home |
| Proximity to the participant | Socially distant | Close | Close | Close | Close | Socially distant | Close | Socially distant |
| Exposure time | None | Short | Prolonged | Short | Prolonged | None | Prolonged | None |
| Aerosolization | None | None | Yes | None | Yes | None | Yes | None |
| PPE Needs | Paper facemask + eye protection + gloves | Paper facemask + eye protection + gloves | N95 + eye protection + gloves + gowns | Paper facemask + eye protection + gloves | N95 facemask + eye protection+ gloves + gowns | ‘  Paper facemask + eye protection + gloves | N95 facemask + eye protection+ gloves + gowns | Paper facemask + eye protection + gloves |
| Overall Risk Score | 2 | 2 | 3 | 2 | 3 | 2 | 3 | 2 |
| Table 5b. Biomarkers - Lab Processing | | | | | | | | |
| Risks | Urine – main study | Whole Blood (finger stick dried blood spots)- main study | Whole Blood (finger stick dried blood spots)- main study | Whole Blood (venous blood draw)- NCI substudy | Buccal cells (buccal scrape)- NCI substudy | Buccal cells (oral rinse)- NCI substudy | Nasal cells (nasal brush)- NCI substudy | Stool (diaper)– ancillary study |
| Aerosolization | none | none | none | none | none | none | none | none |
| PPE Needs | Paper facemask + eye protection + gloves | Paper facemask + eye protection + gloves | Paper facemask + eye protection + gloves | Paper facemask + eye protection + gloves | Paper facemask + eye protection + gloves | Paper facemask + eye protection + gloves | Paper facemask + eye protection + gloves | Paper facemask + eye protection + gloves |
| Overall Risk Score | 2 | 2 | 2 | 2 | 2 | 2 | 2 | 2 |
| *All lab procedures are performed by the staff member without physical contact with a participant or other staff member. | | | | | | | | |

| Table 6. Fetal Growth, Anthropometry and Child Pneumonia Assessment | | | | | |
| --- | --- | --- | --- | --- | --- |
| Risks | Fetal Ultrasound | Anthropometry- Length | Anthropometry- Birth Weight | Hospital visits to screen for pneumonia | Pneumonia home visits |
| Participant | Pregnant woman | Child | Child | Child | Child |
| Location of Procedure | Health center | Home | Health facility (at birth); In home for other visits | Hospital | Home |
| Proximity to the participant* | Close | Close | Close | Close | Vitals performed: close  No vitals performed: socially distant |
| Exposure time | Prolonged | Short | Short | Prolonged | Vitals performed: prolonged  No vitals performed: none |
| Aerosolization | None | Yes- child crying | Yes – child crying | Yes | Vitals performed: Yes, if child cries  No vitals performed: none |
| PPE Needs | Paper facemask + eye protection + gloves | N95 facemask or equivalent respirator + eye protection+ gloves + gowns | N95 facemask or equivalent respirator+ eye protection+ gloves + gowns | N95 facemask or equivalent respirator + eye protection + gloves + gowns | Vitals performed: N95 facemask or equivalent respirator + eye protection+ gloves +/- gowns  No vitals performed: Paper/ cloth facemask |
| Overall Risk | 2 | 3 | 3 | 3 | Vitals performed-3/ No vitals performed -1 |

| Table 7. Vascular Procedures | | | | |
| --- | --- | --- | --- | --- |
| Risks | Bronchial Artery Reactivity Testing (BART) | Carotid Intimal Media Thickness (CIMT) Measurement | Lung Ultrasound | Blood Pressure Measurement |
| Participant | Older adult woman | Older adult woman | Child | Older adult woman |
| Location | Home, health facility, community center | Home, health facility, community center | Health facility | Home, health facility, community center |
| Proximity to the participant | Close | Close | Close | Close |
| Exposure time | Prolonged | Prolonged | Prolonged | Short |
| Aerosolization | None | None | Yes | None |
| PPE Needs | Paper facemask + eye protection + gloves | Paper facemask + eye protection + gloves | N95 facemask or equivalent respirator + eye protection + gloves + gowns | Paper facemask + eye protection + gloves |
| Overall Risk | 2 | 2 | 3 | 2 |
